# ﻿*Rosafuningensis* (Rosaceae), a new species from Yunnan, China

**DOI:** 10.3897/phytokeys.229.101052

**Published:** 2023-07-07

**Authors:** Ling-Na Zheng, Le Luo, Yu-Wei Tang, Chao Yu, Pei-Feng Lyu, Xue-Sen Liu, Qi-Xiang Zhang, Yu-Yong Yang

**Affiliations:** 1 Beijing Key Laboratory of Ornamental Plants Germplasm Innovation & Molecular Breeding, Beijing Laboratory of Urban and Rural Ecological Environment, Engineering Research Center of Landscape Environment of Ministry of Education, Beijing 100083, China Engineering Research Center of Landscape Environment of Ministry of Education Beijing China; 2 National Engineering Research Center for Floriculture, Beijing 100083, China National Engineering Research Center for Floriculture Beijing China; 3 Key Laboratory of Genetics and Breeding in Forest Trees and Ornamental Plants of Ministry of Education, Beijing 100083, China Key Laboratory of Genetics and Breeding in Forest Trees and Ornamental Plants of Ministry of Education Beijing China; 4 School of Landscape Architecture, Beijing Forestry University, Beijing 100083, China Beijing Forestry University Beijing China; 5 Kunming Yang Chinese Rose Gardening Co., Ltd, Kunming 6500871, China Kunming Yang Chinese Rose Gardening Co. Kunming China

**Keywords:** molecular evidence, morphology, new species, *Rosa*, wild germplasm

## Abstract

A new species *Rosafuningensis* and its variant R.funingensisf.rosea, both collected from Yunnan Province, China, are, for the first time, documented and illustrated in this study. Morphological analysis in comparison with two related species in the wild, *R.gigantea* and *R.rubus*, presents distinguishable features through leaf surfaces, inflorescences and the shape of styles. *R.funingensis* leaf surfaces are abaxially villous, purple-red, pale green when mature, adaxially glabrous, dark green; inflorescences solitary or 2–5(7) in corymbose cyme; and styles connate into a column or not, exserted.

## ﻿Introduction

There are about 150–200 species of roses around the world, widely distributed throughout the Northern Hemisphere, with Central and Southwest Asia being the centres of distribution of the genus (Rehder 1951; Ku and Robertson 2003; Quest-Ritson and Quest-Ritson 2003). China has 95 species of the genus *Rosa*, of which 65 species are endemic (Ku and Robertson 2003); there are also 34 varieties, totalling 129 taxa of roses (Liu and Lian 2014). The number of wild rose species in China are found to decrease gradually from the southeast to the southwest and northwest of the country (Yu and Lu 1985). Yunnan is one of the main distribution centres and differentiation centres of *Rosa* (Xu 2001), with 41 species and 17 varieties of wild *Rosa* (Chen and Li 2006).

On 5 April 2018, a unique species of *Rosa* was discovered during an investigation of wild rose resources in Funing County, Wenshan Zhuang and Miao Autonomous Prefecture, Yunnan Province, China. It shared certain morphological characteristics with *R.gigantea* and *R.rubus*, while being distinguished in terms of leaf, inflorescence and shape of styles. Subsequently, this species was introduced to the Kunming South Tropical Garden (Kunming Nanguo Shanhua) Horticulture Technology Co. Ltd., Yunnan Province for further observation and study. After a thorough examination over a period of three years, it was determined that the specific morphological characteristics of this species and its variant were stable, indicating that they were, indeed, new to the *Rosa* genus. In 2021, phylogenetic relationships were analysed after collecting the specimens to confirm their status as a new species within the genus *Rosa*. As a result of this research, the new species was described and named as *Rosafuningensis* L. Luo & Y. Y. Yang. Additionally, a form of this new species was identified and documented as RosafuningensisL. Luo & Y. Y. Yangf.rosea L. Luo & Y. Y. Yang, characterised by its light salmon-pink flowers that fade to white.

## ﻿Materials and methods

### ﻿Field observations, comparative morphology

We studied living plants of the new species in their natural habitats and documented their known distribution ranges. Morphological descriptions and illustrations were based on mature foliage, fresh flowering material and mature fruit of living plants and dried specimens of *R.funingensis* and R.funingensisf.rosea.

### ﻿Phylogenetic analysis

Sixteen taxa of the genus *Rosa*, including *Rosafuningensis* and two outgroups (*Fragariavesca* and *Potentillatanacetifolia*) were used to reconstruct a phylogenetic tree. Sequences of *R.Chinensis* ‘Old Blush’ (sequence number: SRR6175515), *Fragariavesca* (sequence number: SRR12536045) and *Potentillatanacetifolia* (sequence number: SRR8208352) were downloaded from GenBank. The other 14 taxa were selected from six sections. Their complete genomic DNA was extracted from silica-gel-dried leaves using the CTAB method (Porebski et al. 1997) and sequenced using Illumina NovaSeq.

The sequenced data were quality-controlled to obtain clean data. Genome alignment was performed using MINIMAP2 v.2.21. PCR de-duplication and SNP detection were performed using gatk v.4.2.0.0. SNP annotation was performed using ANNOVAR v.2020-06-07. The processed SNP data were analysed in VCFTOOLS v.0.1.17 for Fst analysis (fst-window-size 100000 fst-window-step 10000), thetaΠ analysis (window-pi 100000 window-pi-step 10000) and Tajima’s D analysis (TajimaD 100000). The resulting data were analysed in MEGA11 (Tamura et al. 2021). The evolutionary history was inferred by using the Maximum Likelihood method and the Kimura 2-parameter model (Kimura 1980). The tree with the highest log likelihood (-9601.46) is shown. The percentage of trees in which the associated taxa clustered together is shown next to the branches. Initial tree(s) for the heuristic search were obtained automatically by applying Neighbour-Joining and BioNJ algorithms to a matrix of pairwise distances estimated using the Maximum Composite Likelihood (MCL) approach and then selecting the topology with superior log likelihood value. There were a total of 3560 positions in the final dataset.

## ﻿Results

### ﻿Phylogenetic analysis

**Figure 1.**
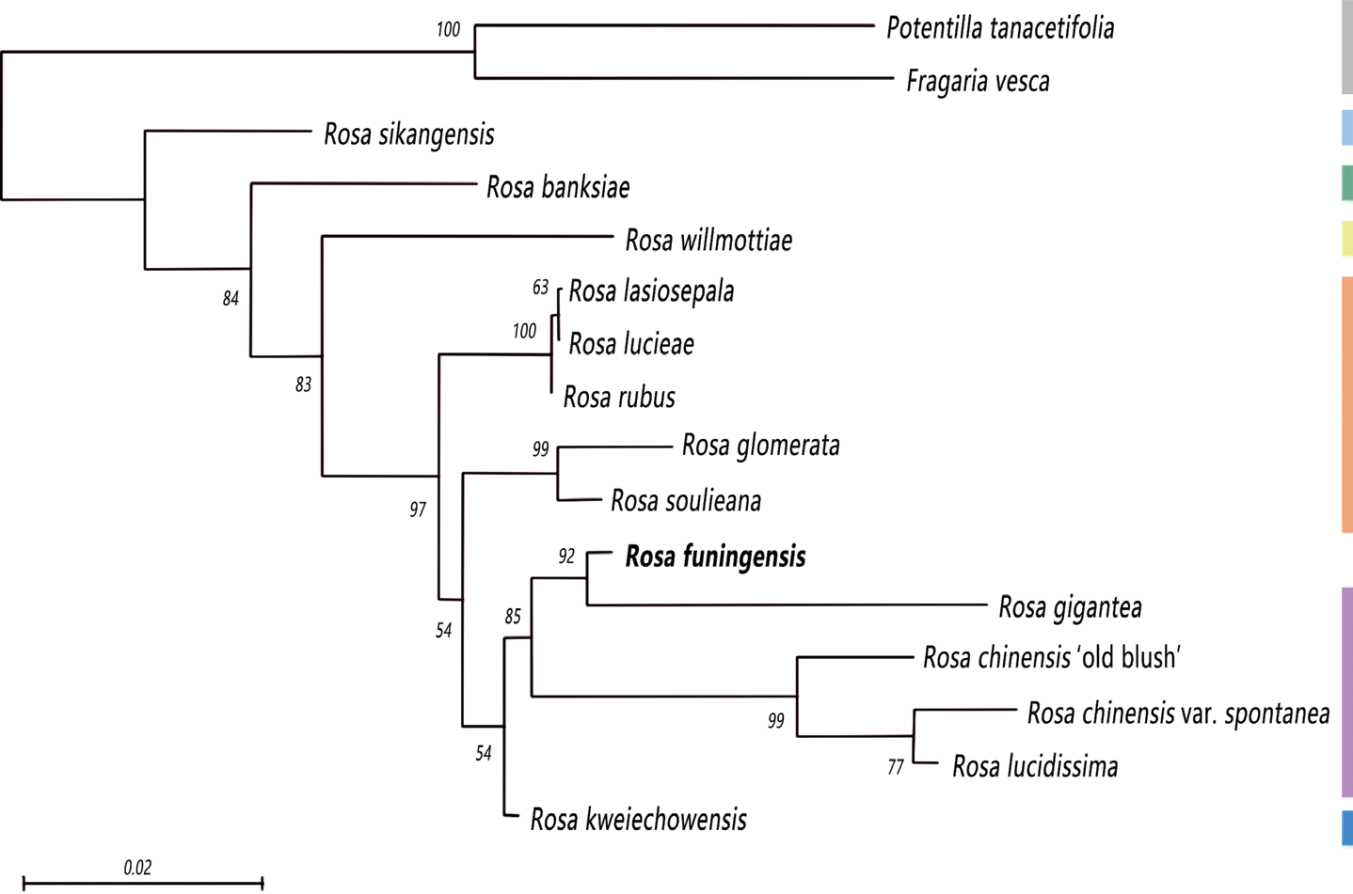
The Maximum Likelihood tree, based on SNPs data. Numbers above branches are ML bootstraps. Grey represents the outgroup, light blue represents sect. Pimpinellifoliae, green represents sect. Banksianae and yellow represents sect. Cinnamomeae. Orange represents sect. Synstylae, purple represents sect. Chinenses and dark blue represents sect. Microphyllae. The new species is shown in bold.

The ML phylogenetic tree (Fig. 1) showed that *Rosaglomerata*, *R.soulieana* (sect. Synstylae) and *R.kweichowensis* (sect. Microphyllae) and all the species of sect. chinenses formed a well-supported clade (purple), with the sister group of *R.lasiosepala*, *R.luciae* and *R.rubus* of sect. synstylae (orange). The putative new species, *R.funingensis*, is placed into a well-supported clade with *R.gigantea* (sect. chinenses) and formed a larger clade with sect. chinenses.

## ﻿Discussion

*Rosafuningensis* is often in association with *R.rubus* and *R.gigantea* in the wild where there are no other members of the genus *Rosa* present. The overlap of the flowering period of *R.rubus* (late March to late April) and *R.gigantea* (March) generates the possibility of natural hybridisation to produce offspring.

Morphologically, *Rosafuningensis* shares similarities with both *R.rubus* and *R.gigantea*, but it is not exactly the same as either one, which provides further evidence that *R.funingensis* may be a natural hybrid. The molecular evolutionary tree also supports this inference.

The discovery of this new species enriches the resources of *Rosa* and provides new materials for interspecific hybridisation. Hybridisation between sections has been a great challenge in rose breeding, making wild Rosa resources not sufficiently exploited (Zhao et al. 2015). The hybridised experiment during 2020–2022 between *R.funingensis* and the wild species of sect. chinenses shows that it is, indeed, a good breeding material. The inclusion of *R.funingensis* as a new germplasm resource for breeding between sect. chinenses and sect. synstylae has the potential to enhance the genetic diversity and improve the breeding outcomes of the genus *Rosa*.

Additionally, during our field research, we also found plants that are similar to *R.funingensis*, but with smaller leaflets (7–9); stipule margin covered with sparsely glandular hairs; flowers showing light salmon-pink at the beginning and turning white at the later stage; hip obovoid. We speculate that these plants may be a form of *R.funingensis*, with an increased number of leaflets and this is currently under observation.

### ﻿Taxonomic treatment

#### ﻿ Rosa funingensis

Taxon classificationPlantaeRosalesRosaceae

L. Luo & Y.Y. Yang sp. nov.

A240F9E9-BBA0-559E-88B9-8B8331D00B08

urn:lsid:ipni.org:names:77322791-1

Figs 2 , 3 , 4

##### Type.

China, Muyang Town, Funing County, Wenshan Zhuang and Miao Autonomous Prefecture, Yunnan Province, 23°25′27″N, 105°21′15″E, 1396 m a.s.l., 31 March 2021, Y. Y. Yang (Holotype BJFC00107680!).

**Figure 2.**
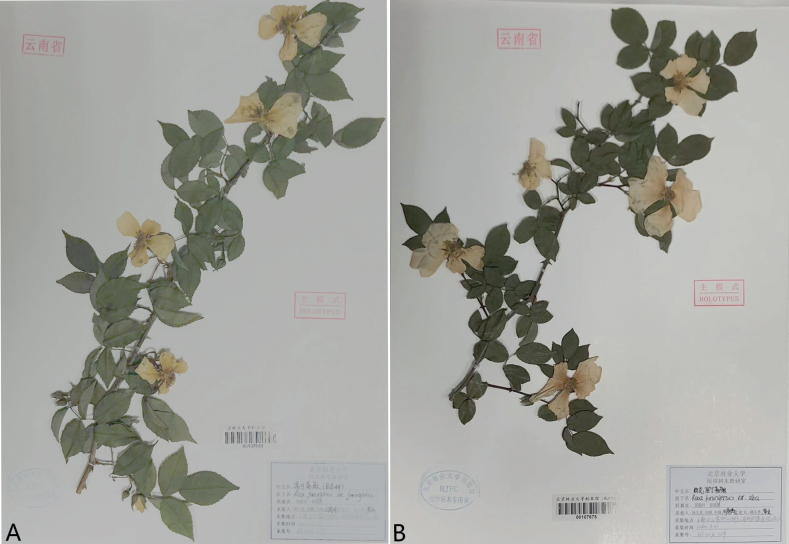
Specimens of *Rosafuningensis* and R.funingensisf.rosea**A***R.funingensis***B**R.funingensisvar.rosea.

##### Diagnosis.

*Rosafuningensis* is mostly similar to *R.gigantea*. However, *Rosafuningensis* differs significantly from *R.gigantea* by having leaves abaxially villous, purple-red, pale green when mature, adaxially glabrous, dark green (vs. both surfaces glabrous), rachis and petiole shortly prickly, glandular hairs and villous (vs. sparsely shortly prickly and glandular pubescent), inflorescences solitary or 3–5(7) in corymbose cyme (vs. solitary or 2 or 3 and fasciculate) and styles connate into a column or not (vs. free) (Table 1).

**Table 1.** Morphological comparisons of *Rosafuningensis*, *R.gigantea* and *R.rubus*.

	*R.funingensis*	*R.gigantea*	*R.rubus*
Leaflet number	5–7	5–9	3–5
Branch	glabrous	glabrous	pubescent when young, glabrous when old
Leaf surface	abaxially pubescent, adaxially glabrous	both surfaces glabrous	abaxially pubescent or glandular, adaxially usually glabrous, rarely pubescent
Rachis and petiole	shortly prickly, glandular hairy and pubescent	sparsely shortly prickly and glandular	pubescent with sparse small curved prickles
Stipule margin	pubescent and glandular	glabrous, or glandular only at free parts	pubescent and glandular
Inflorescence	solitary or 2–5(7) in cyme	solitary	10–25 in cyme
Pedicel	glandular	glabrous or glandular	pubescent and glandular
Flower size (diameter)	7–9 cm	8–9 cm	4–5 cm
Styles	connate into a column or not	free	connate into a column
Hip colour and size (diameter)	yellow, 1.2–1.5 cm	yellow, 2.5–2.8 cm	red, 1.0–1.5 cm

##### Description.

***Rosafuningensis***: Shrubs climbing, new branches 5–6 m long. ***Branchlets*** green, young stems purple-red on sunny side, glabrate; prickles scattered, slightly curved, robust, flat, gradually tapering to broad base. Leaflets including petiole 12–14 cm; stipules mostly adnate to petiole, free parts lanceolate, villous or with short dentate glands at margin, apex acuminate, dry and shrinking when old; rachis and petiole shortly prickly, glandular hairs and villous hairs. ***Leaves*** usually 5–7, often 3 near inflorescence, leaflets obovate or oblong, 3–4 × 2–2.5 cm, apex acuminate, leaves leathery, adaxially glabrous, dark green, abaxially villous, purple-red, pale green when mature, margin with sharp single serrations. ***Inflorescences*** solitary or several in cyme; peduncle with pedicels 1–2 cm, densely glandular hairs; bracts linear, apex acuminate, 1 × 0.3 cm, margin glandular hairy, with prominent mid-vein. ***Flowers*** 7–9 cm in diam.; sepals 5, ovate-lanceolate, abaxially glandular-pubescent, apically caudate, adaxially villous, margin glandular hairs, occasionally linearly divided, reflexed; petals 5, single, white, nearly cordate, apex emarginate, with strong sweet fragrance. Styles connate into a column or not, exserted, light red and the stigma is light yellow. ***Hips*** yellow, subglobose, 1.2–1.5 cm in diam., glabrous. 2n = 14.

##### Phenology.

Flowering in early April, fruiting from July to October.

##### Etymology.

The species epithet refers to Funing County, where the new species was first discovered. The variant with pink flower colour is proposed to be named “Rosafuningensisf.rosea”.

##### Distribution and habitat.

New species are currently known from Funing County, Wenshan Zhuang and Miao Autonomous Prefecture in eastern Yunnan, at elevations between 400 m and 1400 m. They grow on hillsides, roadsides and riversides.

##### Conservation status.

Based on currently available data, the newly-described *Rosafuningensis* species and its variants should be assigned to the ‘Data Deficient’ (DD) category of IUCN (2022). The precise conservation status of the population(s) has not been determined. Further explorations are needed to assess its distribution and conservation status. The known distribution of this species is limited. The type locality of this new species is an unprotected mountainous area. Increasing human activities and habitat destruction may cause a threat to the existence of this rare species.

#### ﻿ Rosa funingensis L. Luo & Y.Y. Yang f. rosea

Taxon classificationPlantaeRosalesRosaceae

L. Luo & Y.Y. Yang f. nov.

5AB10752-5EC6-573C-B019-82FB9D5C5C93

urn:lsid:ipni.org:names:77322792-1

Figs 2 , 3 , 4

##### Type.

China, Muyang Town, Funing County, Wenshan Zhuang and Miao Autonomous Prefecture, Yunnan Province, 1396 m a.s.l., 23°25′27″N, 105°21′15″E, 31 March 2021, Y. Y. Yang (Holotype BJFC00107675!)

**Figure 3.**
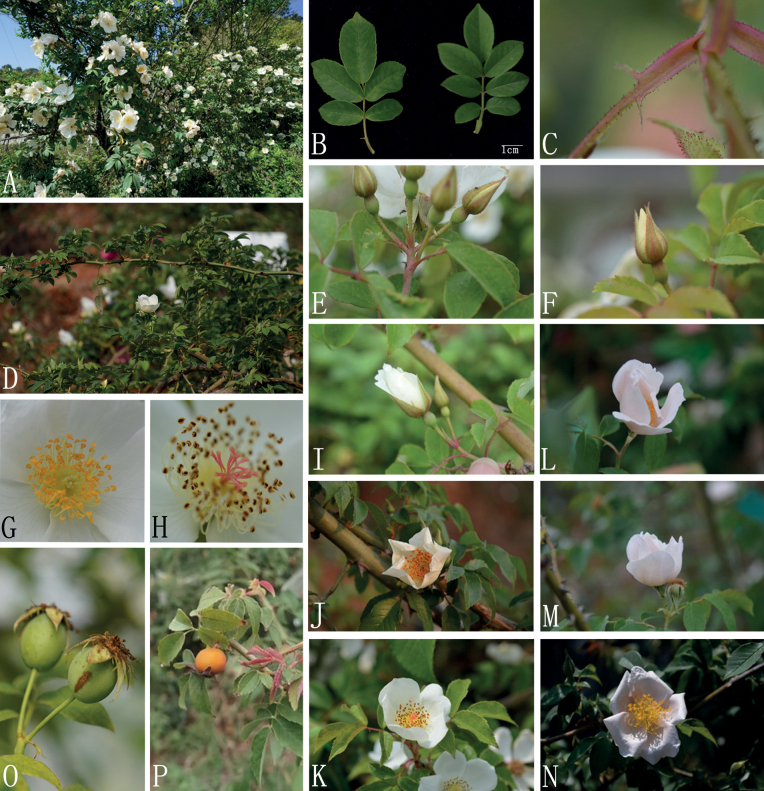
*Rosafuningensis* and R.funingensisf.rosea**A** plant **B** leaves **C** stipule **D** branches and prickles **E, F** two different Inflorescences **G, H** two different styles **I–K** flowering process of *R.funingensis***L–N** flowering process of R.funingensisf.rosea**O, P** hips.

##### Description.

Rosafuningensisf.rosea: Flowers light salmon-pink and fading to white.

**Figure 4.**
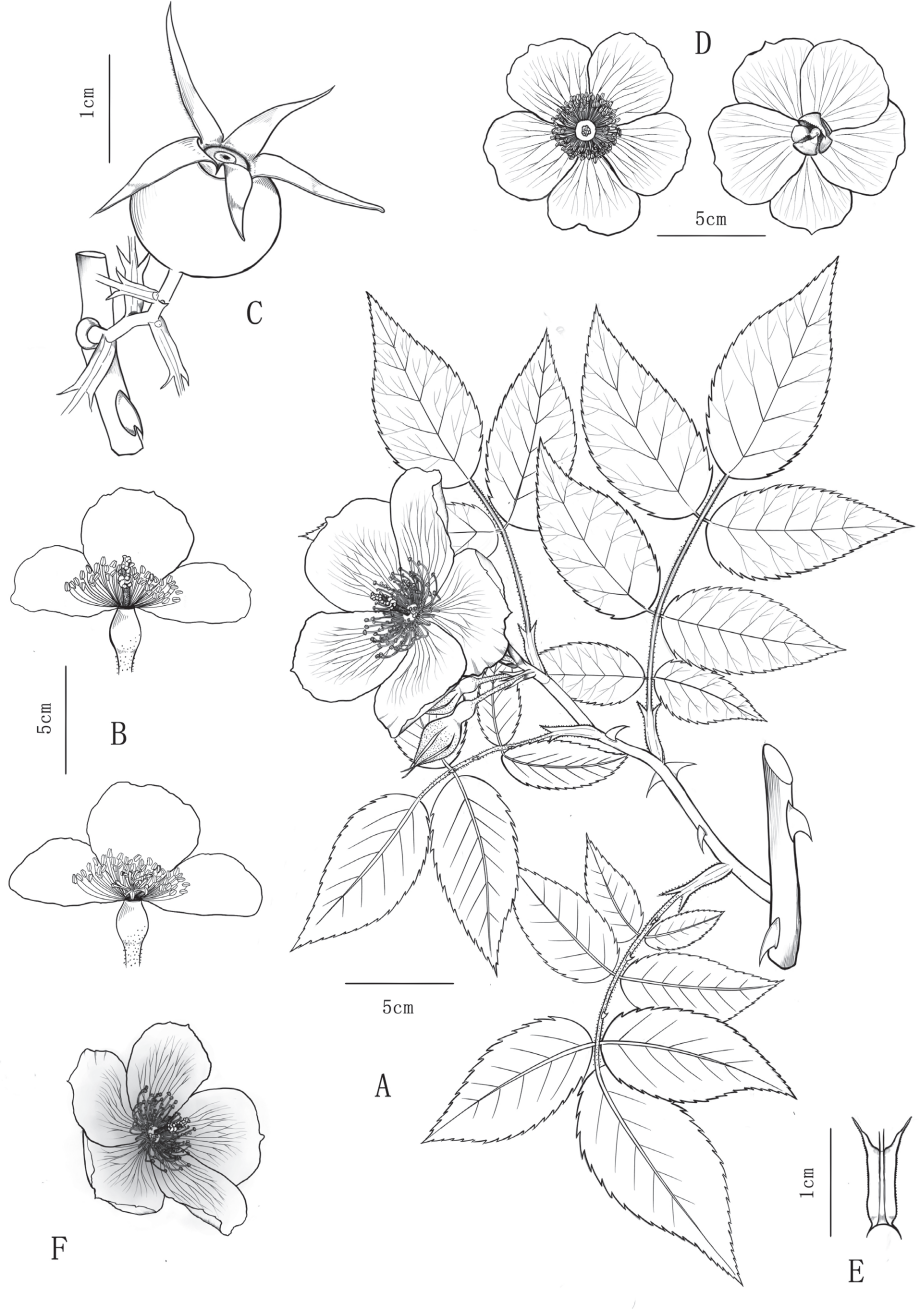
Illustration of *Rosafuningensis* and Rosafuningensisf.rosea**A** whole plant **B** floral anatomy **C** hip **D** flowers of *R.funingensis***E** stipule **F** flowers of R.funingensisf.rosea. Drawn by Y. W. Tang.

##### Etymology.

The variant with pink flower colour is proposed to be named “Rosafuningensisf.rosea”.

### ﻿Identification key to taxa in R.sect.Chinenses and *Rosarubus*

**Table:** 

1	Styles connate into a column; sepals pinnately lobed; mature hips red	***R.rubus***
–	Syles free or connate into a column; sepals often entire, occasionally pinnately lobed; mature hips yellow	**2**
2	Young stems glabrous, leaflets 5–7–(9); flowers always open wide and flat and are floppy in full bloom; hips globose or depressed-globose	**3**
–	Young stems pubescent or glabrous; leaflets 3 – 5 – (7); flowers hardly completely open, often high-centred (bowl-shaped) in full bloom; hips ovoid, obovoid or globose	**4**
3	Leaves glabrous; stipule margin glabrous, or only glandular at free parts; flowers solitary; styles free	***R.gigantea***
–	Leaves abaxially pubescent; stipule margin pubescent and glandular; flowers solitary or 2–5(7) in cyme; styles connate into a column or not	***R.funingensis***
4	Young stems pubescent; leaflets 3–(5)	***R.lucidissima***
–	Young stems glabrous; leaflets (3)–5–(7)	**R.chinensisvar.spontanea**

## Supplementary Material

XML Treatment for Rosa funingensis

XML Treatment for Rosa funingensis L. Luo & Y.Y. Yang f. rosea

## ﻿Additional information

### Author ORCIDs

Ling-Na Zheng https://orcid.org/0000-0002-3850-5413

Le Luo https://orcid.org/0000-0002-8143-1931

Yu-Wei Tang https://orcid.org/0000-0001-9722-2299

Chao Yu https://orcid.org/0000-0002-8096-5333

Pei-Feng Lyu https://orcid.org/0000-0001-9969-5356

Xue-Sen Liu https://orcid.org/0000-0002-0240-2551

Qi-Xiang Zhang https://orcid.org/0000-0001-5690-0065

Yu-Yong Yang https://orcid.org/0000-0002-4134-6847

### Data availability

All of the data that support the findings of this study are available in the main text or Supplementary Information.

## Supplementary materials

Supplementary material 1 Comparisons of *R.funingensis*, *R.gigantea*, and *R.rubus* Data type species data https://binary.pensoft.net/file/874443This dataset is made available under the Open Database License (http://opendatacommons.org/licenses/odbl/1.0/). The Open Database License (ODbL) is a license agreement intended to allow users to freely share, modify, and use this Dataset while maintaining this same freedom for others, provided that the original source and author(s) are credited.Ling-Na Zheng
