# Supplementary material for: ﻿Rosafuningensis (Rosaceae), a new species from Yunnan, China
Source: PhytoKeys. 2023 Jul 7;229:61–70. doi: 10.3897/phytokeys.229.101052 (PMC10349306; doi:10.3897/phytokeys.229.101052)
Supplement: Supplementary material 1 — Comparisons of R.funingensis, R.gigantea, and R.rubus [file phytokeys-229-061_article-101052__-s001.docx]

|  |  | *Rosa funingensis* | | *Rosa gigantea* | | *Rosa rubus* | |
| --- | --- | --- | --- | --- | --- | --- | --- |
| 1 | leaflets number | Leaflets 5-7, often 3 near the inflorescence | 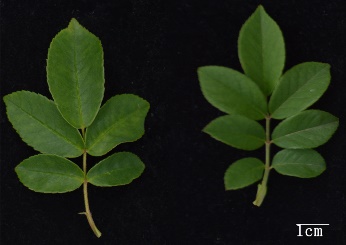 | Leaflets 5–9 | 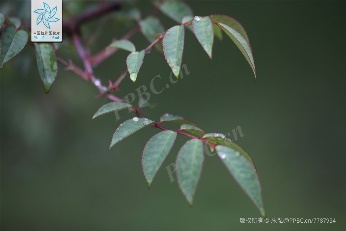 | Leaflets 3 or 5 | 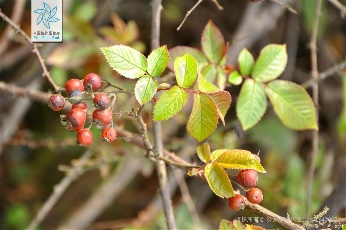 |
| 2 | Branch | glabrous | 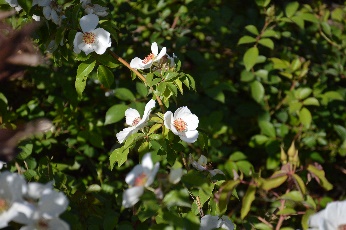 | glabrous | 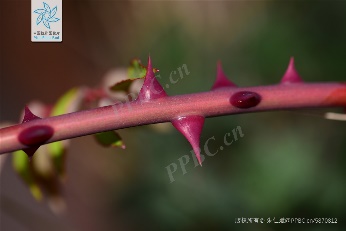 | pubescent when young, glabrate when old | 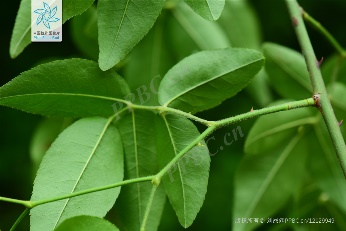 |
| 3 | Leaves surface | Abaxially villous, purple-red, pale green when mature, adaxially glabrous, dark green | 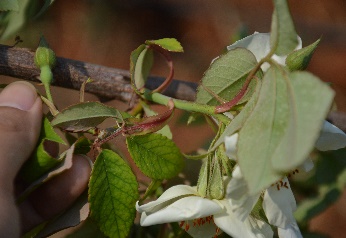 | Both surfaces glabrous | 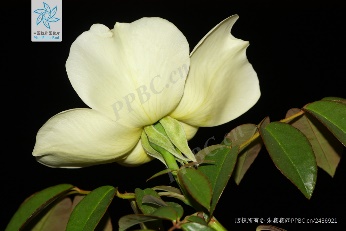 | Abaxially densely or sparsely pubescent or glandular, adaxially usually glabrous, rarely pubescent | 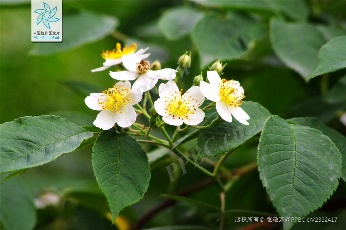 |
| 4 | rachis and petiole | shortly prickly, glandular hairs and villous | 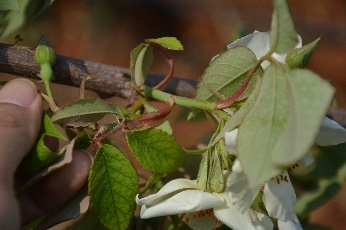 | sparsely shortly prickly and glandular pubescent | 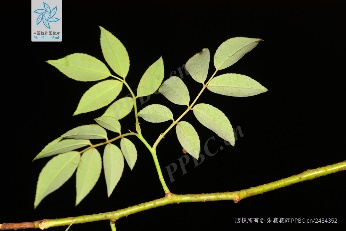 | pubescent, with sparse small hooked prickles | 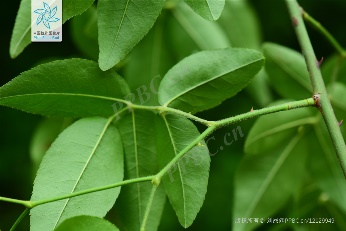 |
| 5 | stipules | free parts lanceolate, margin villous and sparsely short dentate glands. | 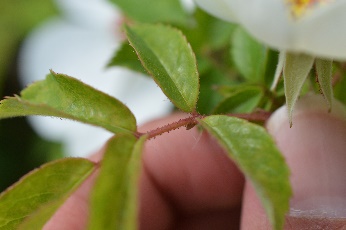 | free parts auriculate, glabrous, glandular at margin or only at base | 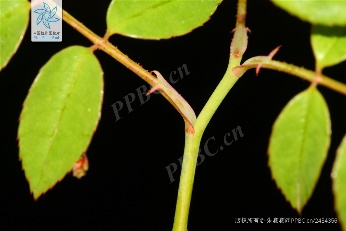 | free parts lanceolate, pubescent, margin entire, often glandular | 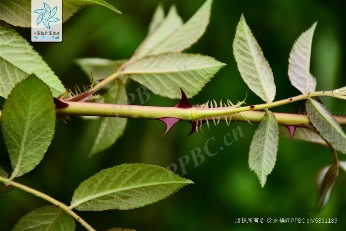 |
| 6 | Inflorescences | Solitary or 3-5(7) in corymbose cyme | 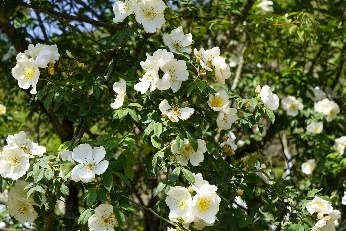 | solitary, or 2 or 3 and fasciculate | 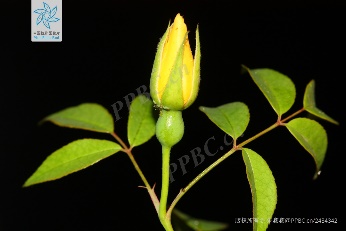 | 10-25 in a paniculate corymb | 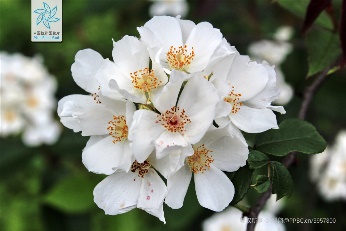 |
| 7 | pedicel | densely glandular hairs | 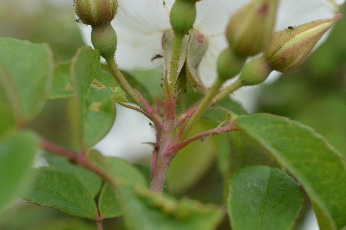 | glabrous or glandular pubescent | 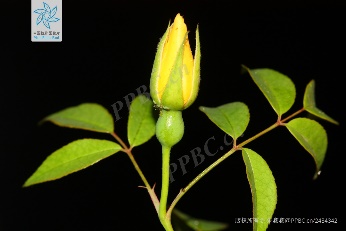 | pubescent and glandular pubescent | 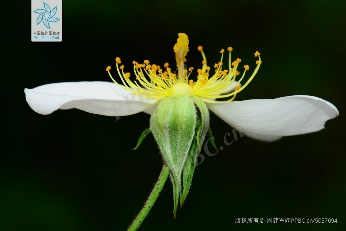 |
| 8 | Flower size (diameter) | 7-9 cm |  | 8-9 cm |  | 4-5 cm |  |
| 9 | Petal shape | nearly cordate, apex emarginate | 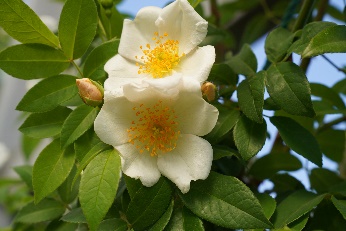 | obovate, base cuneate, apex emarginate | 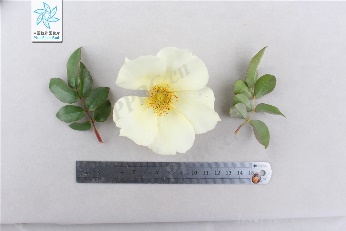 | obovate, base broadly cuneate, apex emarginate | 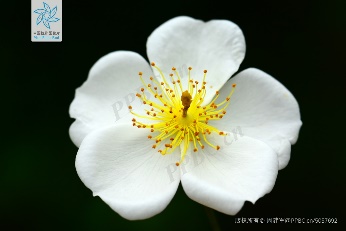 |
| 10 | Styles shape | Styles connate into column at the beginning, free at the later stage | 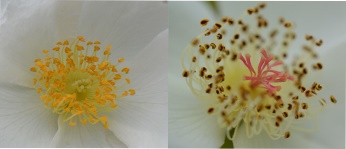 | Styles free | 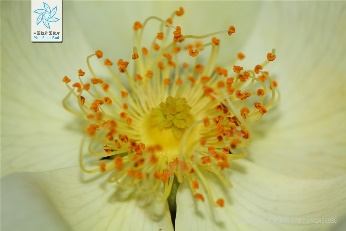 | Styles connate into the column | 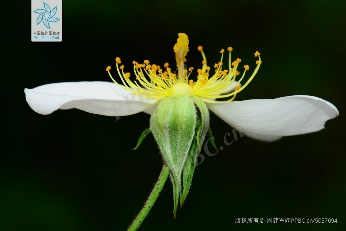 |
| 11 | Hip shape and size (diameter) | yellow, 1.2-1.5 cm | 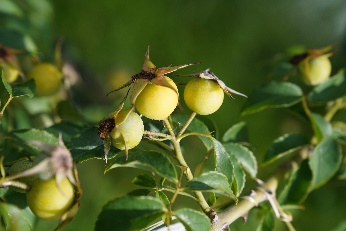 | yellow, 2.5-2.8 cm | 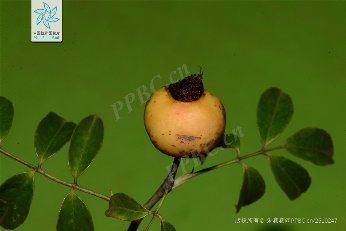 | red, 1.0-1.5 cm | 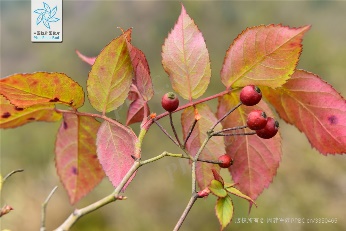 |
